# Parasite control practices on Swedish horse farms

**DOI:** 10.1186/1751-0147-49-25

**Published:** 2007-09-26

**Authors:** Eva Osterman Lind, Erik Rautalinko, Arvid Uggla, Peter J Waller, David A Morrison, Johan Höglund

**Affiliations:** 1 Department of Parasitology (SWEPAR), Swedish University of Agricultural Sciences and National Veterinary Institute, SE-751 89 Uppsala, Sweden; 2 Department of Psychology, Uppsala University, P.O. Box 1225, SE-751 42 Uppsala, Sweden

## Abstract

**Background:**

Virtually all horses are infected with helminth parasites. For some decades, the control of parasites of Swedish horses has been based on routine treatments with anthelmintics, often several times per year. Since anthelmintic resistance is becoming an increasing problem it is essential to develop more sustainable control strategies, which are adapted to different types of horse management. The aim of this study was to obtain information on practices used by Swedish horse owners for the control of endoparasites.

**Methods:**

A questionnaire with 26 questions about management practices and parasite control routines was posted to 627 randomly selected horse establishments covering most types of horse management in Sweden.

**Results:**

The response rate was good in all categories of respondents (66–78%). A total of 444 questionnaires were used in the analyses. It was found that virtually all horses had access to grazing areas, usually permanent. Generally, pasture hygiene was infrequently practiced. Thirty-six percent of the respondents clipped or chain harrowed their pastures, whereas weekly removal of faeces from the grazing areas was performed by 6% of the respondents, and mixed or rotational grazing with other livestock by 10%. The number of anthelmintic treatments per year varied from 1–8 with an average of 3.2. Thirty-eight percent considered late autumn (Oct-Dec) to be the most important time for deworming. This finding, and an increased use of macrocyclic lactones in the autumn, suggests a concern about bot flies, *Gasterophilus intestinalis*. Only 1% of the respondents stated that faecal egg counts (FEC) were performed on a regular basis. The relatively high cost of FEC analyses compared to purchase of anthelmintics was thought to contribute to the preference of deworming without a previous FEC. From the study it was evident that all categories of horse owners took advice mainly from veterinarians.

**Conclusion:**

The results show that routines for endoparasite control can be improved in many horse establishments. To increase the knowledge of equine endoparasite control and follow the recommendations for how to reduce the spread of anthelmintic resistance, a closer collaboration between parasitologists and veterinary practitioners is desirable.

## Background

The horse is host to a great number of gastrointestinal helminths, of which nematodes of the family Strongylidae, the roundworm *Parascaris equorum *and the cestode *Anoplocephala perfoliata *are the most important. These parasites are ubiquitous and have been recognised as significant causes of clinical disease in horses. A previous Swedish study has shown that 2–3 year-old horses on stud farms, particularly in the south of the country, often shed high numbers of strongyle eggs [1]. Although the occurrence of the most pathogenic species, *Strongylus vulgaris*, has decreased markedly during the past 3 decades, eggs of this nematode were still found in samples from 14% of the investigated farms [1]. Furthermore, studies of *A. perfoliata *have revealed a high prevalence of this parasite in horses of all ages [2].

The officially estimated number of horses in Sweden is 283,100 [3], of which the great majority are leisure and sports horses. Generally, the control of endoparasites of Swedish horses relies largely on anthelmintics, which are easy to administer and rarely cause negative clinical side effects. Unfortunately, excessive use of anthelmintic drugs has led to the development of resistance in populations of cyathostomins worldwide [4]. Moreover, cases of decreased susceptibility of *P. equorum *to macrocyclic lactones were reported recently from The Netherlands and Canada [5,6]. In Sweden, benzimidazole resistance in cyathostomins was shown on 96% of the farms [7], and recently occurrence of pyrantel resistance has been recognised [8]. Despite its widespread use for 20 years there are still no reports of resistance to ivermectin in horse strongyles. Nevertheless, in parallel to the situation in e.g. sheep trichostrongylids, it is believed that resistance against macrocyclic lactones will appear also in equine parasites [9].

In view of emerging anthelmintic resistance it is important to develop more sustainable control strategies, which are adapted to different types of horse management. The aim of the present questionnaire was to obtain information in order to describe the current routines and practices used by Swedish horse owners for the control of endoparasites. No previous work of this type has been performed in Sweden.

## Methods

A questionnaire on management practices and parasite control routines was developed and tested on 60 horse owners in a pilot study prior to commencing the main study. Subsequently the form was slightly revised, and the final version comprised 26 questions, of which 3 were open-ended and the rest closed (Table 1). In order to include the main types of horse establishments in the study, names of respondents were obtained from the following lists:

**Table 1:** Summary of questions in the questionnaire

*Question*	*Reply options*
*Type of establishment*	
Type of horses^1^	Trotters; riding horses; cold-blooded trotters; ponies; working horses; Thoroughbreds; Icelandic
Type of establishment^1^	Stud; livery; trotting stable; riding school; small private farm
Total number of horses	
Number of individuals younger than 5 years	

*Pasture management*	
Access to grazing area	No access; pasture summertime; all-year-round paddocks with grass
Do you practice mixed or rotational grazing?	Yes; no
Grazing areas and procedures practiced^1^	Permanent pastures; fertilisers used; aftermath grazed; ploughing and re-seeding every 2^nd^–3^rd ^year; rotation between clean plots; pasture clipping/harrowing; grazing areas are part of crop rotation system
Do you remove manure from the grazing areas?	No; at least once per week; at least once per month; at least once per year

*Use of anthelmintic drugs*	
Do you de-worm your horses?	Yes; no
Person responsible for de-worming	Horse owners; manager; staff; veterinarian; other
How is the dosage calculated?	By guessing the horse weight; by measuring the horse weight; by age; one tube of paste per animal
Which drug brands were used at different months for the last 12 months^2^?	
When did you de-worm horses <5 yrs for the last 12 months?^3^	
When did you de-worm horses >4 yrs for the last 12 months?^3^	
Do you de-worm regularly against tapeworm?	No/sporadically; yes with double dose of pyrantel; yes with praziquantel; do not know
Which of the following drugs have you used during the past 24 months^1^?	All anthelmintic drugs and brands registered for horse are shown to the responder
Which routines are practiced on your farm?^1^	De-worming of new horses; individual de-worming programmes; horses sharing pasture de-wormed together; horses of the same age de-wormed together; all horses on the farm de-wormed together
What time of the year do you think is the most important for de-worming?	Jan-March; April-June; July-Sept; Oct-Dec
Do you look for parasites in faeces after deworming?	Yes; no

*Attitudes and encountering of parasite problems*	
Have you seen bot flies/eggs for the last 2 years?	Yes; no
What is your attitude regarding anthelmintic usage? State the importance on a 1–5 scale.	Environmental aspects; cost; resistance; targeted de-worming; alternatives to anthelmintic drugs
Have you had a faecal sample analysed from a healthy horse?	No; once; several times; regularly
For how many years have you been active with horses?	
Do you consider helminth infection as a problem in horses? State the magnitude of the problem on a 1–5 scale.	
Have you experienced problems caused by endoparasites?	Yes; no
How important are the following sources of information for you? State the importance on a 1–5 scale.	Information on the package of the drug; other horse owners; Apoteksbolaget (Swedish Pharmacy Chain); books; internet; veterinarian; horse magazines

^1 ^Possibility to choose more than one option

^2 ^Respondents were to combine anthelmintic trade names and months of the year in a chart

^3 ^The months were to be marked by the respondent

• Individuals who had horses insured by Agria, the principal Swedish insurance company for horses;

• Riding clubs that were members of Svenska Ridsportförbundet (the Swedish Riding Association);

• Professional trainers with an A-licence from Svenska Travsportens Centralförbund (the Swedish Trotting Association);

• Members of the Svenska hingsthållarföreningen (Swedish Stallion Holders' Association);

• Stallion studs that were members of Avelsföreningen för Svenska Varmblodiga Hästen (the Swedish Warmblood Association).

From each of these lists, establishments were selected randomly using slips of paper or random numbers generated by the computer. The questionnaire was finally posted to 627 horse establishments (out of approximately 56 thousand) in 2003. To maximise the number of respondents, a book on horses was rewarded those who completed the questionnaire. In addition, reminder letters were posted to those who had not returned the questionnaire after 2 and 4 weeks.

Based on information from the completed questionnaires, respondents were classified into the following types of establishments:

• Stud farms – at least 10 horses; several foals bred every year;

• Livery yards – mainly horses older than 4 years; different horse owners;

• Trotting stables – often large establishments with a high proportion of young horses being trained; a great turnover of horses;

• Riding schools – usually horses older than 4 years; generally short grazing periods;

• Small private – establishments with a small number of horses; often one owner.

A few establishments were multipurpose stables. These were classified according to what seemed to be the principal type.

Data summaries and descriptive statistical analyses were calculated using Microsoft Excel X for Mac Service Release 1 (Microsoft Corporation). The frequency data were analysed with log-likelihood contingency (for 4 variables) or goodness-of-fit (for 1 variable) tests with Williams correction for continuity (G-tests; [10]), using Microsoft Excel X for Mac Service Release 1 (Microsoft Corporation). The continuous data were analysed by 2-factor orthogonal analyses of variance, using SYSTAT 9 for Windows (SPSS Inc, Chicago IL, USA). For all three analyses one factor was the type of establishment, with the other factor being either horse age, anthelmintic feature or information source. It all cases it is the interaction hypothesis that is of interest. In order to assess the effect of possible non-normality on the analyses, they were repeated using non-parametric 2-factor orthogonal Kruskal-Wallis tests [11]. For all three analyses the same qualitative result was obtained from both the parametric and non-parametric tests; and so only the parametric results are presented here (i.e. means and interaction F-values). All tests were considered to be statistically significant at P < 0.05.

## Results

### Response

In total, replies were obtained from 447 (71%) of the establishments. However, 3 respondents were excluded because less than half of the questions had been answered (Table 2). The response rate was good in all types of farms (66–78% depending on type). For each question the amount of missing data varied from 0 to 12%. In all cases, our reported percentages are the percentage of those who responded to the question.

**Table 2:** Data on establishments that returned the questionnaire

	*Stud farms*	*Livery yards*	*Trotting stables*	*Riding schools*	*Small private*	*Total*
Number of respondents	51	44	112	117	120	444
%	12%	10%	25%	26%	27%	
Response rate	70%	72%	66%	78%	72%	71%
Mean number of horses (range)	53 (10–200)	15 (3–70)	31 (4–130)	35 (9–90)	6 (1–36)	26
Proportion <5 yrs (range)	50% (13–100)	21% (0–80)	68% (20–100)	4% (0–24)	22% (0–100)	31%

### Pasture management

Overall, 97% of the respondents (n = 429) stated that their horses had access to grazing areas. On all stud farms and small private farms horses had access to grazing areas, whereas 89% of the trotting stables, 98% of the livery yards and 99% of the riding schools kept horses on grazing areas.

Most establishments, particularly livery yards, stated that the pastures were permanent, i.e. used solely for horses for several consecutive years (Table 3). Pasture clipping, or chain harrowing, were the most common pasture hygiene procedures undertaken; 36% of the respondents clipped or harrowed their pastures. Twenty-six percent of the establishments carried out more than one of the pasture improvement procedures shown in Table 3. On stud farms a majority (74%) used more than one of these procedures.

**Table 3:** Percentage of respondents that carried out different pasture management procedures

*Pasture procedures*	*Stud farms*	*Livery yards*	*Trotting stables*	*Riding schools*	*Small private*	*Total *n = 429
Permanent pastures	61%	93%	83%	87%	83%	83%
Aftermath grazed	16%	12%	5%	9%	15%	11%
Ploughing and re-seeding	20%	5%	8%	4%	17%	11%
Rotation between clean plots	24%	7%	4%	12%	13%	11%
Pasture clipping/harrowing	76%	28%	35%	25%	33%	36%
Pastures part of crop rotation system	43%	7%	6%	3%	10%	11%

**Figure 1:**
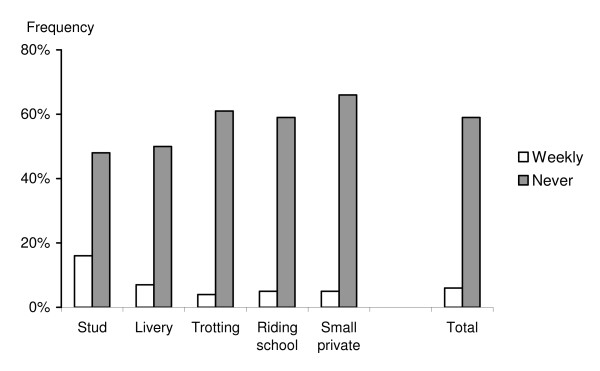
Percentage of respondents who removed manure weekly or never did it. Other options are not displayed in the figure.

Forty-one percent of the respondents (n = 179) stated that they removed faeces from the grazing areas (Fig 1). Of those, 6% stated that they did it at least once per week, whereas 11% did it at least once per month and 24% once per year. In addition, only 10% (n = 47) practiced mixed or rotational grazing with other animal species, such as cattle or sheep. The proportion of respondents that did practice mixed or rotational grazing with other livestock species varied from 4% (trotting stables) to 18% (stud farms).

### Use of anthelmintic drugs

**Figure 2:**
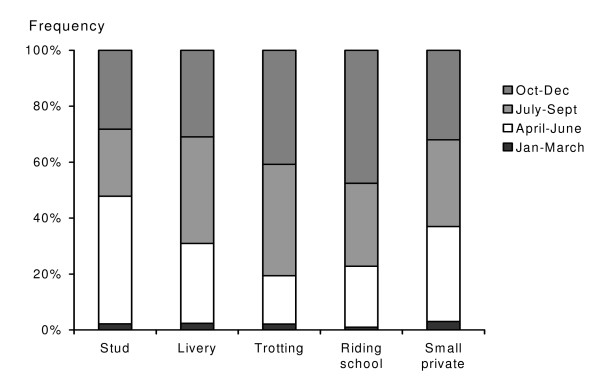
Times of the year considered to being the most important for de-worming (n = 387).

Virtually all respondents (99.5%, n = 442) stated that they de-wormed their horses. Only 8 respondents (2%) considered winter (Jan-March) as the most important time for de-worming. This was significantly (G = 28.3, df = 3, P < 0.001) fewer than for other times of the year (Fig 2).

To calculate the dosage of anthelmintic, 13% out of 441 stated that they measured the horse's weight (girth tape or scale), 67% guessed the weight, and the remainder, particularly trotting stables, administered one tube of anthelmintic paste per animal. Eighty-two percent of 439 respondents stated that they de-wormed all horses on the farm at the same time and 38% de-wormed new horses on arrival at the farm. Nearly all (98%, n = 431) respondents stated that treatments were performed by either the horse owners or farm managers. Ten farms (2%), of which 6 were trotting stables, stated that anthelmintic treatments were carried out by a veterinarian. The number of anthelmintic treatments per year varied from 1–8 with an average of 3.2. Generally (F = 13.10, df = 4, P < 0.001), horses younger than 5 years were treated more frequently (3.5 times) than older horses (3.0 times). This was particularly evident for stud farms, where young horses were treated 4.4 times per year and older horses 3.3 times. Stud farms and trotting stables treated horses of all ages more frequently than did other types of establishments. In livery yards, riding schools and small private establishments, the adult horses were treated 2.6–2.8 times per year, and for livery yards and small private establishments there was no difference in treatment frequency between young and old horses.

**Figure 3:**
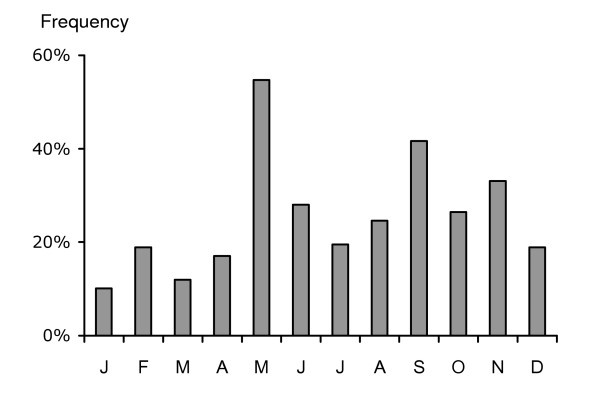
Frequency distribution of anthelmintic treatments performed during the previous 12 months. Note that numbers add up to more than 100 because the horses are dewormed more than once per year.

Anthelmintic treatments were performed during all months of the year, but most respondents stated that they used anthelmintics less during the winter months (Jan-March; Fig 3). Those who de-wormed their horses in the winter were mainly respondents on stud farms and trotting stables. The percentages of establishments that de-wormed in May (55%) and September (42%) were significantly (G = 284.9, df = 11, P < 0.001) higher than during other times of the year.

**Figure 4:**
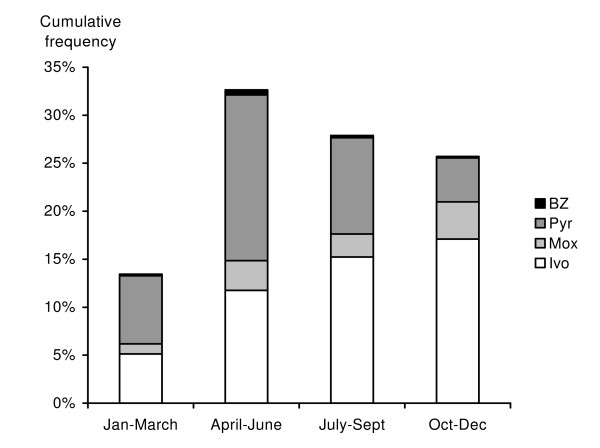
Usage of different anthelmintic drugs at various times of the year. BZ – benzimidazoles; Pyr – pyrantel; Mox – moxidectin; Ivo – ivermectin.

Ivermectin and pyrantel were the most commonly used drugs. The benzimidazoles were used in only 0–2% of the treatments (Fig 4). There was a significant (G = 127.8, df = 9, P < 0.001) seasonally related difference between the use of pyrantel and ivermectin drugs: pyrantel dominated in the spring and ivermectin in the autumn. Less than half of the respondents (41%, n = 180) had observed bot flies or their eggs during the previous 24 months.

The majority of the respondents (65%, n = 272) had used 2 different classes of anthelmintic drugs (excluding praziquantel) during the previous 12 months; 9% (n = 38) had used 3 classes and the remaining had used one class. Furthermore, it was stated by 50% of the respondents that they de-wormed with pyrantel at 38 mg/kg bodyweight against tapeworms, whereas 1% used praziquantel. Most of the treatments against tapeworms were performed in May, although all months of the year were represented. Thirty-nine percent did not de-worm against tapeworm, and 10% did not know if they did.

### Attitudes to and encountering of parasite related problems

Some features regarding attitude to anthelmintic treatment are presented in Table 4. Respondents from all types of farms considered anthelmintic resistance to be of great importance (mean 4.6 out of 5), whereas a demand for faecal analysis prior to de-worming was of low importance (mean 2.3). The cost of anthelmintic compounds was listed as most important for riding schools. Generally (F = 2.40, df = 16, P = 0.001), establishments with trotters considered that all features specified in Table 4 were of less importance than did other types of establishments.

**Table 4:** Mean scores regarding anthelmintic treatment related features, This was classified on a scale 1–5, where 1 was irrelevant and 5 very important

*Classified features*	*Stud farms*	*Livery yards*	*Trotting stables*	*Riding schools*	*Small private*	*Total (St.dev) *n = 429
Environmental aspects	3.1	3.3	2.8	3.3	3.2	3.1 (1.2)
Cost of drug	3.3	3.0	2.3	3.5	2.7	2.9 (1.3)
Anthelmintic resistance	4.7	4.5	4.3	4.7	4.6	4.6 (0.8)
Demand for faecal egg counts prior to de-worming	2.3	2.8	2.0	2.5	2.4	2.3 (1.0)
Alternatives to anthelmintics	3.5	3.3	2.9	3.4	3.5	3.3 (1.1)

Only 1% (n = 5) of the respondents sent faecal samples for parasitological analysis on a regular basis. Sixty-eight percent (n = 302) had never sent a sample and the remaining 31% (n = 135) had sent samples occasionally once or several times. The proportion of respondents that had sent samples several times was significantly (G = 64.2, df = 12, P < 0.001) higher among trotting stables than other types of establishments.

**Figure 5:**
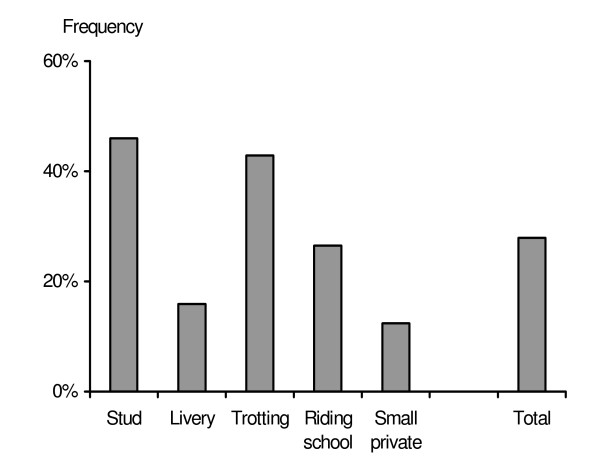
The proportion of respondents (n = 444) who had personal experience of helminth related problems in their horses.

Seventy-two percent of the respondents had never experienced any problems that they could associate with helminth infections (Fig 5). Still, they scored the problem of helminth infections in horses as a mean of 2.5 on a 1–5 scale. Significantly (G = 39.0, df = 4, P < 0.001) higher proportions of respondents on stud farms and trotting stables stated that they had experienced problems with helminth infections than those on other types of farms.

In general, information regarding parasites and parasite control seemed to be of significance. All respondents considered the veterinarian to be the most important information source, whereas information gained from the internet and other horse owners were the least important (Table 5). With the exception of veterinarians, information sources were generally scored lower by trotting stables and stud farms than by other categories of respondents (F = 3.06, df = 24, P < 0.001).

**Table 5:** Mean scores regarding information on parasites and control, This was classified on a scale 1–5, where 1 was totally unimportant and 5 very important

*Information source*	*Stud farms*	*Livery yards*	*Trotting stables*	*Riding schools*	*Small private*	*Total (St.dev) *n = 437
The package of the drug	3.8	4.3	4.0	4.1	4.3	4.1 (1.0)
Other horse owners	2.7	3.2	2.5	2.8	3.2	2.9 (1.1)
Swedish Pharmacy Chain (Apoteksbolaget)	3.0	3.9	2.9	3.5	3.8	3.4 (1.3)
Books	3.5	3.9	3.1	3.6	3.6	3.5 (1.1)
Internet	2.5	3.4	2.3	2.9	2.9	2.7 (1.3)
Veterinarians	4.5	4.7	4.7	4.8	4.5	4.7 (0.7)
Horse magazines	4.2	3.9	3.4	4.1	3.7	3.8 (1.1)

## Discussion

The overall response rate was good for all types of farms. However, valid conclusions may be drawn only if the returned questionnaires are representative of all Swedish horse establishments. We consider this likely to be true. First, a representative range of establishments was targeted for the survey. Second, the response rate was high across all categories of establishments. Third, we attempted to independently verify the accuracy of our data. For example, anthelmintic treatments as described by the respondents to the survey were compared to statistics on the sales of anthelmintic drugs for horses, and they were found to be in agreement.

As 97% of the respondents stated that their horses had access to grazing areas, usually permanent pastures, it can be concluded that the conditions on Swedish horse farms are favourable for the transmission of pasture-borne endoparasites. In general, pasture hygiene was infrequently practised. However, stud farms appeared to apply pasture hygiene practices to a greater extent than respondents on other types of farms, which suggests an awareness of contaminated pasture as the main source of infection for important endoparasites. Pasture clipping and/or chain harrowing obviously were the most commonly used procedures, especially on stud farms (76%). The main reason for clipping the pasture is to improve the re-growth of grass and to remove weeds. During the process of clipping, the faeces will disperse over the pasture, sometimes after it has been broken up by a cutter, which can be attached to the clipper. Chain harrowing is primarily performed to prevent high levels of infective larvae on pasture. As a consequence of pasture clipping or chain harrowing during hot and dry weather, the infective larvae will die from desiccation [12]. Still, only 6% of the respondents removed faeces at least once per week, which is considerably lower than has been reported from the UK, where 49% of the respondents stated that they collected faeces at least once per week [13]. Furthermore, 90% of the establishments in the present study did not practice mixed or rotational grazing with other livestock, although the benefit of such grazing management is often highlighted in parasite control recommendations. Thus, it appears that Swedish horse owners are less inclined, or have less possibility, to mix or rotate grazing with sheep or cattle than are horse owners in Denmark (18%), England (44%) or Ireland (71%) [14-16].

Practically all respondents de-wormed their horses, and only occasionally did a veterinarian perform these treatments. The exceptionally low use of benzimidazoles is in accordance with statistics from the Swedish Pharmacy Chain on the sales of anthelmintics for horses in Sweden. Ivermectin and pyrantel were the most frequently used drugs. Interestingly, late autumn was considered to be the most important time of the year for de-worming, although the frequency distribution of performed treatments showed that more farms had de-wormed in May (spring) than in any other month. The reason why the respondents regarded late autumn as the most important time for de-worming is most likely due to awareness of the bot fly, *Gasterophilus intestinalis*, which had been evident on 41% of the farms. Also, the increased use of macrocyclic lactones in late autumn indicates the concern among Swedish horse owners about *G. intestinalis*. In order to preserve the efficacy of anthelmintic drugs a number of recommendations have been developed, namely: (1) minimising the number of doses, (2) slow rotation between different drug classes, (3) correct dosing, (4) effective treatment of new individuals before introduction to the herd, and (5) regular monitoring for resistance [17]. Slow rotation of drug classes on an annual or biennial basis implies that *G. intestinalis *control is excluded in the years when macrocyclic lactones are not used. The same principle has to be applied to treatment with pyrantel against the tapeworm *Anoplocephala perfoliata*. However, administration of more than one drug class per year on 65% of the farms, and more than two classes on 9%, suggests that the recommendation of a slow rotation of drug classes has not been fully adopted in Sweden.

Compared to Ireland [15] and the UK [13], Swedish horse owners appear to de-worm less frequently. The most intensive anthelmintic control programmes were found on stud farms and trotting stables, i.e. establishments that typically accommodate a great number of young horses. However, it can be speculated that the treatment frequency for adult horses in Sweden is unnecessarily high. In a previous study of strongyle egg output in Swedish horses it was found that 59% of the animals older than 4 years excreted as few as 0–100 eggs per gram of faeces [1]. Moreover, in livery yards and small private establishments there was no difference in the number of treatments between young and adult horses, which either suggests overuse of anthelmintics in adult horses or the opposite in young horses.

The vast majority (82%) of respondents stated that all horses on their farm were treated at the same time. However, only 38% of the respondents treated new horses before introduction to the herd. In the future, treatment of new horses, preferably with macrocyclic lactones, obviously needs to be emphasised by veterinarians in order to avoid the introduction of resistant parasite strains.

In the control of endoparasites of livestock in general, increased attention is being paid to targeted selective anthelmintic treatments based on prior faecal egg count (FEC) monitoring [18]. For equines, studies have shown that targeted treatments may reduce the total number of treatments and thereby also the selection pressure for anthelmintic resistance [19-21]. Application of targeted treatments, as well as the recommendation to monitor regularly for anthelmintic resistance, require both the involvement of a veterinarian and laboratory analyses of faecal samples. In this study, only 1% of the respondents stated that they consign samples for FEC analysis on a regular basis, with the majority never sending faecal samples from healthy horses. Thus, it appears that Swedish horse owners perform FECs less frequently than do horse owners in Ireland, where 28% of the owners performed FECs at least once per year [15]. Reasons for a low frequency of FEC analysis could be: (1) in Sweden the analysis of a faecal sample is considerably more expensive than the dose of anthelmintic, (2) the cost of anthelmintics does not appear to be of decisive importance to the horse owners, and (3) lack of motivation, as it was generally considered that parasites are a minor problem, and 72% of the respondents had no personal experience of parasite related problems in their horses. Consequently, the respondents also considered that faecal sampling prior to de-worming was of minor importance. Methods alternative to anthelmintic treatment were considered as being of average importance. However, respondents on all types of farms considered anthelmintic resistance to be an important, or very important, issue. Altogether, these results indicate that Swedish horse owners are concerned about anthelmintic resistance but they lack the knowledge, or motivation, as how to delay its development.

Finally, it was evident in all categories of horse owners that veterinarians were the most important source of information regarding parasite control, whereas the internet still seems to be of minor importance. Hence, to increase horse owners' knowledge of endoparasites and parasite control, the most appropriate way would be to further educate veterinarians. This could be achieved by courses, leaflets and articles, but additionally a closer collaboration between parasitologists and veterinary practitioners would be beneficial for all parties concerned.

## Authors' contributions

EOL was responsible for the design of the study, performance of the pilot study, the distribution of the questionnaire and writing the draft of the manuscript. ER contributed in the design of the study and in the formulation of questions. AU, PJW and JH participated in the design of the study and helped to word the final version of the manuscript. DAM did most of the data analyses and the interpretation of these.
